# ﻿A new gorgonian *Pseudopterogorgiananjiensis* sp. nov. (Cnidaria, Octocorallia, Gorgoniidae) from the Nanji Islands, China

**DOI:** 10.3897/zookeys.1213.126841

**Published:** 2024-09-27

**Authors:** Tingzai Sun, Yu Xu, Kuidong Xu, Shun Chen, Shangwei Xie, Zifeng Zhan

**Affiliations:** 1 Laboratory of Marine Organism Taxonomy and Phylogeny, Qingdao Key Laboratory of Marine Biodiversity and Conservation, Shandong Province Key Laboratory of Experimental Marine Biology, Institute of Oceanology, Chinese Academy of Sciences, Qingdao 266071, China Institute of Oceanology, Chinese Academy of Sciences Qingdao China; 2 University of Chinese Academy of Sciences, Beijing 100049, China University of Chinese Academy of Sciences Beijing China; 3 Southern Marine Science and Engineering Guangdong Laboratory (Zhuhai), Zhuhai 519082, China Laboratory for Marine Biology and Biotechnology, Qingdao Marine Science and Technology Center Qingdao China; 4 Laboratory for Marine Biology and Biotechnology, Qingdao Marine Science and Technology Center, Qingdao 266237, China Southern Marine Science and Engineering Guangdong Laboratory Zhuhai China; 5 National Engineering Research Center of Marine Facilities Aquaculture, Zhejiang Ocean University, Zhoushan 316022, China Zhejiang Ocean University Zhoushan China; 6 Nanji Islands National Marine Nature Reserve Administration, Wenzhou 325400, China Nanji Islands National Marine Nature Reserve Administration Wenzhou China

**Keywords:** Anthozoa, COI, Malacalcyonacea, morphology, mtMutS, new species, phylogeny, *Pseudopterogorgiananjiensis*, taxonomy, 28S rDNA

## Abstract

Members of the genus *Pseudopterogorgia* Kükenthal, 1919 are widely distributed in shallow water of the Indo-West Pacific. During an investigation of benthic biodiversity in the subtidal zone surrounding the Nanji Islands in the East China Sea, two specimens of *Pseudopterogorgia* were collected and described as a new species based on an integrated morphological-molecular approach. *Pseudopterogorgiananjiensis***sp. nov.** is most similar to *P.fredericki* Williams & Vennam, 2001 in the irregular branching form and indistinct scaphoids, but differs by the coenenchymal sclerite content of distinct capstans and a few warty spindles and radiates (vs. mostly warty spindles and a few capstans), and a purplish colony (vs. white, pink to deep rose). Molecular phylogenetic analyses, based on the mtMutS-COI gene sequences, delineated a monophyletic clade encompassing all assessed *Pseudopterogorgia* species. Within this clade, *P.nanjiensis***sp. nov.** showed a close phylogenetic affinity with both *P.fredericki* and *P.australiensis* Ridley, 1884.

## ﻿Introduction

Octocorals fulfill a pivotal ecological function, with their three-dimensional configurations enhancing the structural intricacy and variety of reefs (Poliseno 2016; Lau et al. 2020). Gorgonian corals in octocoral animal forests offer nourishment and habitat for a variety of interrelated organisms (Sánchez 2016). Among these, the genus *Pseudopterogorgia* Kükenthal, 1919 represents a rare group of shallow-water octocorals found in the Indo-West Pacific (Williams and Chen 2012). The members of this genus are characterized by pinnate or irregular branching structures, absence of anastomoses, and C-shaped or scaphoid-like sclerites present in the coenenchyme (Kükenthal 1919; Bayer 1961; Grasshoff and Alderslade 1997). To date, this genus has seven valid species, all of which are distributed in the Indo-West Pacific (Williams and Chen 2012; WoRMS Editorial Board 2024).

During the investigation of benthic biodiversity in the subtidal zone of the Nanji Islands on the Chinese coast of the East China Sea, two specimens of *Pseudopterogorgia* were collected. They are described as a new species *Pseudopterogorgiananjiensis* sp. nov. based on morphological and phylogenetic analyses. The phylogenetic placement of *P.nanjiensis* sp. nov. within the genus is further explored.

## ﻿Material and methods

### ﻿Specimen collection and morphological examination

**Figure 1.**
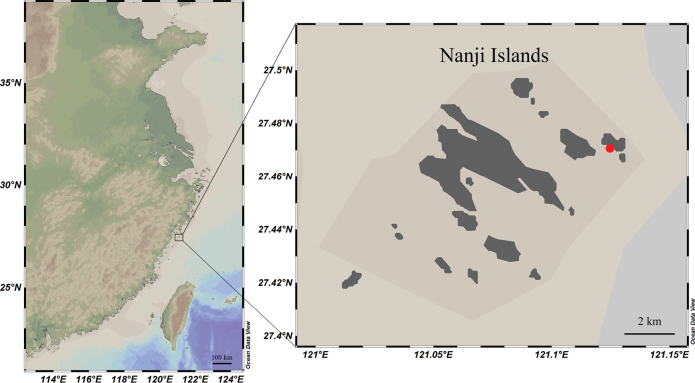
Sampling site (red dot) off the coast of the Nanji Islands in the East China Sea.

Two specimens were obtained by scuba diving from the subtidal zone of the Nanji Islands (27°28.53'N, 121°08.13'E) on the Chinese coast of the East China Sea during investigation of the benthic biodiversity in 2022 and 2023 (Fig. 1). The specimens were photographed in situ using a Nikon D850 before being sampled and on board using a Canon EOS 5D Mark IV, and then stored in 75% ethanol after collection.

A stereo dissecting microscope (Zeiss SteREO Discovery. V20) was used to examine the general morphology and anatomy of the specimens. Sclerites of the polyps and branches were isolated by digesting the tissues in sodium hypochlorite and subsequently rinsed thoroughly with deionized water. To investigate the ultrastructure of polyps and sclerites, they were air-dried, mounted on carbon double adhesive tape, and coated with Pd/Au for scanning electron microscope (SEM) observation. SEM scans were obtained by using a TM3030Plus SEM at 15 kV, and the optimum magnification was selected for each type of sclerite. The terminology used in this study follows Bayer et al. (1983).

The type specimens of the new species have been deposited in the Marine Biological Museum of Chinese Academy of Sciences (MBMCAS) at Institute of Oceanology, Chinese Academy of Sciences, Qingdao, China.

### ﻿DNA extraction and sequencing

Genomic DNA was extracted from the polyps of each specimen using the DNeasy Blood and Tissue Kit (Qiagen, Hilden, Germany) following the instructions. Specific regions of the mitochondrial *mutS* homolog (mtMutS) and cytochrome *c* oxidase subunit I (COI), as well as an approximately 800-nt fragment of the 28S nuclear ribosomal gene (28S rDNA), were selected for phylogenetic analysis. For the amplification of mtMutS and COI, the primer pairs AnthoCorMSH (5’-AGGAGAATTATTCTAAGTATGG-3’; Herrera et al. 2010) and Mut-3458R (5’-TSGAGCAAAAGCCACTCC-3’; Sánchez et al. 2003); COI8414-F (5’-CCAGGTAGTATGTTAGGRGA-3’) and HCO2198 (5’-TAAACTTCAGGGTGACCAAAAAATCA-3’) were utilized, respectively. Sequencing of 28S rDNA was performed using the primers 28S-Far (5’-CACGAGACCGATAGCGAACAAGTA-3’) and 28S-Rar (5’-TCATTTCGACCCTAAGACCTC-3’) (McFadden and van Ofwegen 2012). Amplifications and sequencing of the markers followed Xu et al. (2021).

### ﻿Genetic distance and phylogenetic analyses

The sequences of Gorgoniidae species and two species of Eunicellidae as an out-group were downloaded from GenBank (Table 1). The sequences were aligned using MAFFT v.7 (Katoh and Standley 2013) with the L-INS-I algorithm. Genetic distances of mtMutS, COI and 28S between species/populations were calculated with MEGA 6.0 using Kimura 2-parameter model (Tamura et al. 2013).

**Table 1.** The sequences used in this study. Newly sequenced species are in bold.

Species	Voucher Number	GenBank Accession Numbers		
		mtMutS	COI	28S rDNA
***Pseudopterogorgiananjiensis* sp. nov.**	MBM287892	PP558212	PP556906	PP572455
***Pseudopterogorgiananjiensis* sp. nov.**	MBM287893	PP558213	PP556907	PP572456
*Pseudopterogorgiaaustraliensis*	–	AY268442	–	–
*Pseudopterogorgiafredericki*	CAS:118507	JX152766	–	–
*Pseudopterogorgiarubrotincta*	CAS:155043	JX152768	–	–
*Leptogorgiaalba*	-/HMG71/MECN Ant0018	AY268452	HG917083	KX721241
*Leptogorgiacuspidata*	-/HMG97/MCZ:7002	AY268450	HG917088	KX767433
*Leptogorgiamariarosae*	MECN Ant0012	KX721193	KX721174	KX721231
*Leptogorgiaobscura*	Ant 0066/Ant 0066/BEIM 0080	KX767321	KX767383	KX721248
Leptogorgiacf.palma	USNM:1516865/-	ON109715	MW401657	–
*Eugorgiadaniana*	MECN Ant0033	KX721208	KX721189	KX721246
*Pacifigorgiairene*	Ant 0044/Ant 0044/MNCN 2.04/1174	KX767346	KX767406	KX767449
*Gorgoniaflabellum*	-/-/RMNH Coel.40827	AY126427	GQ342418	JX203708
*Phyllogorgiadilatata*	–	AY126428	–	–
*Antillogorgiabipinnata*	-/ABBAC034/RMNH Coel.40828	GQ342499	MK153463	JX203712
*Adelogorgiaosculabunda*	MZUCR:2496_OCT0088	MF579541	–	–
Psammogorgiacf.arbuscula	HMG38/HMG15/HMG38	HG917043	HG917055	LT221092
*Chromoplexauramarki*	–	KX904972	KX904954	–
*Callistephanus* sp.	USNM:1606569	ON109735	–	–
*Eunicellatricoronata*	RMNH Coel.40814	JX203795	JX203853	JX203707
*Complexummonodi*	CSM-SEN3/CSM-SEN4/CSM-SEN4	KP036906	KP036909	KP036912

Phylogenetic analyses were conducted on the 28S rDNA and mtMutS-COI datasets. The phylogenetic frameworks were established using PhyloSuite v.1.2.3 (Zhang et al. 2020; accessible at http://phylosuite.jushengwu.com), a comprehensive platform for phylogenetic analysis. ModelFinder v.2.2.0 (Kalyaanamoorthy et al. 2017) was employed to identify the optimal evolutionary models based on the Bayesian Information Criterion (BIC). HKY+F+I and TIM3e+I were selected as the best-fit substitution models for the mtMutS-COI and 28S rDNA alignments, respectively. Maximum likelihood (ML) trees were inferred using IQ-TREE v.2.2.0 (Nguyen et al. 2015) with 1000 standard bootstraps. Following Hillis and Bull (1993), ML bootstrap values were categorized into low (<70%), moderate (70–90%), and high (≥90%) confidence levels. Bayesian inference (BI) phylogenies were reconstructed using MrBayes v.3.2.7a (Ronquist et al. 2012) with dual parallel runs, each consisting of 10,000,000 generations and a burn-in phase that discarded the initial 25% of sampled data. Following Alfaro et al. (2003), the Bayesian posterior probabilities < 0.95 and ≥ 0.95 were considered as low and high, respectively.

## ﻿Results

**Phylum Cnidaria Hatschek, 1888**

**Subphylum Anthozoa Ehrenberg, 1834**

**Class Octocorallia Haeckel, 1866**

**Order Malacalcyonacea McFadden, van Ofwegen & Quattrini, 2022**

**Family Gorgoniidae Lamouroux, 1812**

**Genus *Pseudopterogorgia* Kükenthal, 1919**

### ﻿ Pseudopterogorgia nanjiensis

Taxon classificationAnimaliaCnidariaGorgoniidae

Sun, Xu & Zhan sp. nov.

A084F647-A3E2-57D8-96D5-29E71EF9322A

https://zoobank.org/37902653-255A-46C8-9867-7C8DDC045BA3

**Figure 2.**
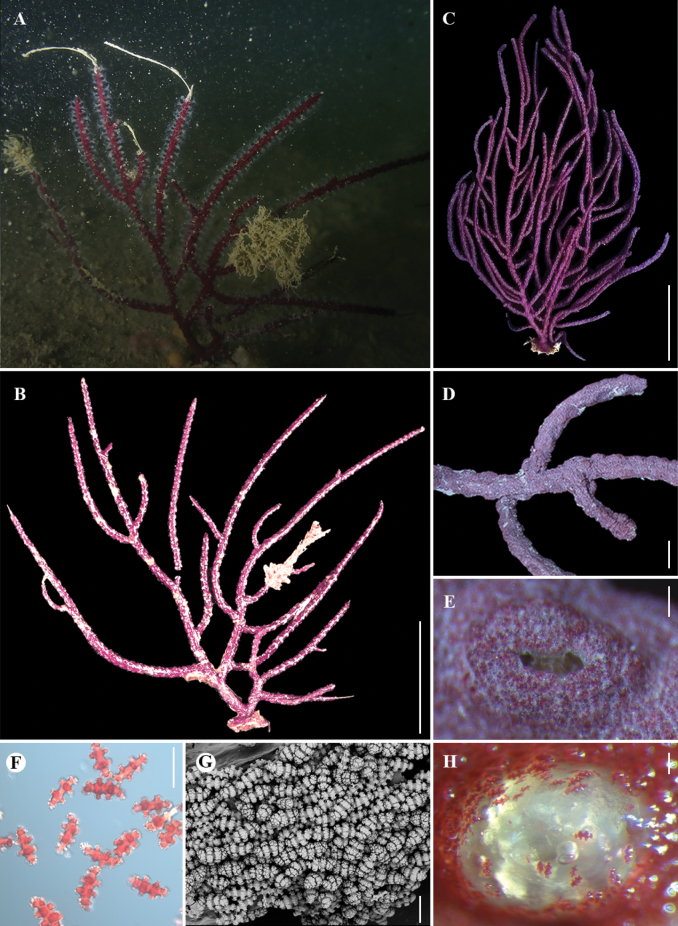
The external morphology and polyps of *Pseudopterogorgiananjiensis* sp. nov. **A** holotype in situ **B** holotype after collection **C** paratype after fixation **D** part of branch under a light microscope **E** single polyp aperture under a light microscope **F** sclerites under a light microscope **G** coenenchyme under SEM**H** single dissolved polyp aperture under a light microscope. Scale bars: 2 cm (**B**); 3 cm (**C**); 2 mm (**D**); 0.2 mm (**E**); 0.1 mm (**F, G, H**).

**Figure 3.**
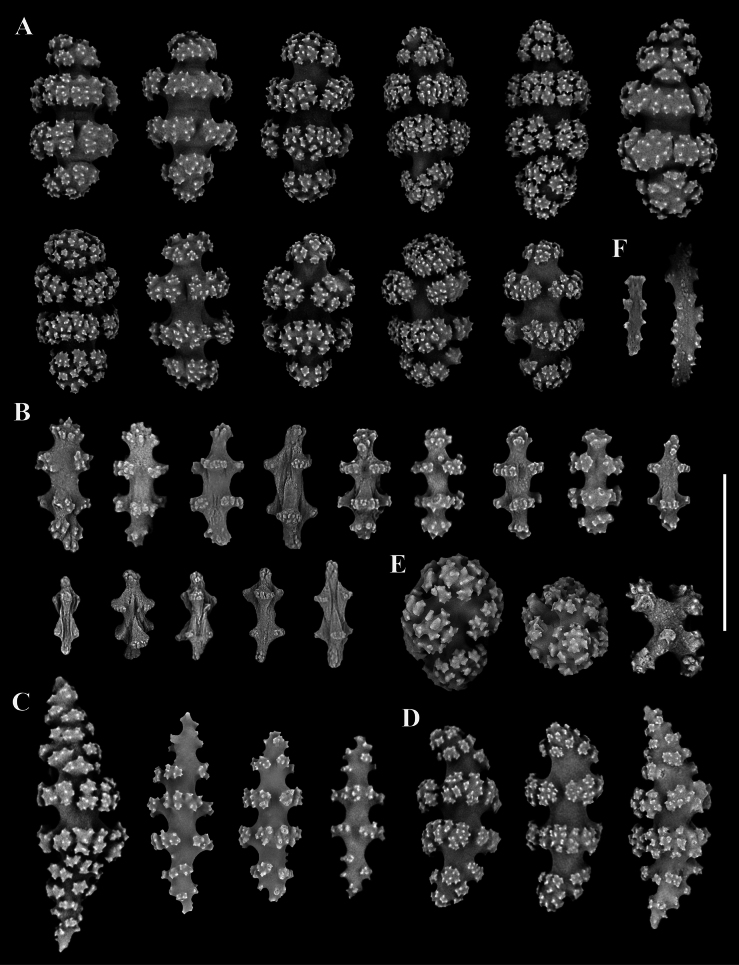
The sclerites of *Pseudopterogorgiananjiensis* sp. nov. **A** capstans **B** radiates and immature sclerites **C** warty spindles **D** scaphoids **E** tuberculate spheroids and cross **F** flat rodlets. Scale bars: 0.1 mm (all in the same scale).

Figs 2 , 3 , Table 2

#### Material examined.

***Holotype***: MBM287893, China-Zhejiang Prov. • Nanji Islands; 27°28.53'N, 121°08.13'E; 11 May 2023. ***Paratype***: MBM287892, China-Zhejiang Prov. • Nanji Islands; 27°28.53'N, 121°08.13'E; 14 m; 11 May 2022.

#### Diagnosis.

Colony non-pinnate and purplish. Branches divided dichotomously and irregularly, nearly in one plane. Polyp retracted and forming a small and oval-shaped protrusion. Sclerites in polyps small flat rodlets, colorless and rare to absent in numbers. Sclerites in coenenchyme red, mostly capstans, a few radiates and warty spindles, and rare scaphoids. Capstans have two whorls of tubercles and blunt ends with irregular arrangements of complex tubercles. Radiates and immature sclerites with two whorls of projections, spindles with 4–8 whorls of tubercles, and scaphoids with similar ornamentation of tubercles on both convex and concave sides.

#### Description.

Holotype upright and nearly planar but not pinnate, about 70 mm in length (Fig. 2B). Holdfast nearly round, about 8 mm long and 7 mm wide at maximum. Trunk cylindrical, about 3 mm in length before first branch and 2 mm in diameter at base, divided into two primary stems, one with three secondary branches and the other with five secondary branches. Branches divided dichotomously and irregularly, nearly in one plane (Fig. 2D). Distances between adjacent branches up to 20 mm. Branches usually cylindrical in the proximal part of the colony, and became flattened in the distal part of the colony. Terminal branchlets slender and a little curved, typically measuring 20–60 mm in length and 1–2 mm in width.

Polyp retracted, forming small and oval-shaped protrusions with a recessed orifice (Fig. 2E), measuring on average 1.00 mm in length and 0.65 mm in width. Each polyp’s orifice appears as a tiny and pale yellowish slit aligned lengthwise. Polyps usually closely and irregularly spaced on the branches and sparse at the basal part of the colony, some of them arranged into two opposing longitudinal rows on the terminal branchlets. Distance between polyps no more than 4 mm.

Coenenchyme covered with dense sclerites, while the polyps sclerites rare to absent (Fig. 2G, H). Sclerites in coenenchyme red with various shapes, arranged in two inconspicuous layers without a clear boundary, including abundant capstans, rare indistinct scaphoids, and occasional tuberculate spheroids and crosses in outer layers, a few radiates and warty spindles in inner layers. These sclerites range in length from 0.05 mm to 0.18 mm, but mostly less than 0.12 mm long (Figs 2F, 3A–E). Among them, capstans have two whorls of tubercles, up to 0.13 mm long, and blunt ends with irregular arrangements of complex tubercles (Fig. 3A). The radiates and immature sclerites with two whorls of projections and often blunt ends, some of them covered with shallow longitudinal grooves on surface, measuring 0.05–0.09 mm (Fig. 3B). The warty spindles with 4 to 8 transversely-aligned whorls of tubercles, up to 0.18 mm in length (Fig. 3C). The scaphoids, not distinctly developed and resembling curved spindles, with 4–8 noticeable whorls of tubercles, up to 0.14 mm long (Fig. 3D). The convex and concave sides have a similar degree of fine ornamentation on the whorls of tubercles, and there seems to be minimal variation in the tubercles between the two sides with those on the convex sides potentially being slightly shorter. Tuberculate spheroids and crosses occasionally present in coenenchyme, are up to 0.08 mm and 0.06 mm long, respectively (Fig. 3E). Small rodlets in polyps flat and colorless, rare in numbers and only found in a few polyps, covered with sparse warts on the edges, up to 0.09 mm (Fig. 3F).

Holotype purplish in situ and after collection, and became deep purplish red after fixation.

#### Variation of paratypes.

The two specimens exhibited a high degree of morphological concordance, with discrepancies primarily noted in colony size and branching density. Paratype irregularly branched, about 140 mm in length and 90 mm in width (Fig. 2C). The main trunk before first branch about 5 mm in length and 2 mm in diameter at base. The terminal branchlets 30–100 mm long and 1–2 mm wide. The oval-shaped protrusions formed by retracted polyps about 1.00 mm long and 0.75 mm wide. The warty spindles in coenenchyme up to 0.16 mm long.

#### Type locality.

The subtidal zone of Nanji Islands with water depth of 14 m.

#### Etymology.

Named after the type locality Nanji Islands.

#### Distribution and habitat.

Known only from the subtidal zone of the Nanji Islands on the Chinese coast of the East China Sea with a water depth of 14 m. Colony attached to a rocky substrate. The water temperature was 18 °C and the pH was 8.13.

#### Remarks.

Given the non-uniform nature of these variations, they are treated as manifestations of intraspecific variability. *Pseudopterogorgiananjiensis* sp. nov. is most similar to *P.fredericki* Williams & Vennam, 2001 in the irregular branching form and indistinct scaphoids, but differs by the sclerite forms and sizes (almost capstans, a few warty spindles and radiates, and rare indistinct scaphoids and small rodlets, mostly less than 0.12 mm vs. almost warty spindles and a few scaphoids, mostly more than 0.12 mm) and the purplish colony (vs. white, pink to deep rose) (Williams and Vennam 2001; Figs 2, 3, Table 2). *Pseudopterogorgiananjiensis* sp. nov. is also analogous to *P.formosa* Nutting, 1910 in the irregular branching form, but distinct from *P.formosa* by the sclerite forms (capstans, spindles, radiates, and scaphoids vs. spindles, double heads, and scaphoids) (Nutting 1910; Table 2). In addition, the new species can be readily distinguished from the remaining congeners by its irregular branching form (vs. pinnate or lateral; Table 2).

**Table 2.** Comparison of morphological characters of *Pseudopterogorgia* species (based on Williams and Vennam 2001). “-” means unavailable data.

Characters/Species	*P.nanjiensis* sp. nov.	*P.fredericki*	*P.formosa*	*P.australiensis*	*P.luzonica*	*P.oppositipinna*	*P.rubrotincta*	*P.torresia*
Branching	irregular	irregular	irregular	pinnate/ plumose	lateral	pinnate to irregular	pinnate	pinnate/ sparse
Sclerite forms	capstans, radiates, warty spindles, scaphoids, flat rodlets	elongated spindles, some shorter and more robust spindles, scaphoids	small or double or girdled spindles, a few double heads, scaphoids	straight or slightly curved spindles, eight radiates, scaphoids	girdled spindles, scaphoids	elongated spindles, scaphoids	rough spindles, scaphoids	warty spindles, quadriradiate, scaphoids
Scaphoids	indistinct	indistinct	some distinct	some distinct	mostly distinct	mostly distinct	some distinct	some distinct
Colony color	purplish	white, pink to deep rose	dark bright pink to carmine	yellow, orange or deep crimson	bright red	brown to amber-yellow.	orange yellow with red line	pale yellow
Sclerites color	red or colorless	reddish or colorless	red, carmine	yellow or red	red	purple-red or crimson red	red	–
Maximum sclerite length	0.18 mm	0.23 mm	0.17 mm	0.17 mm	0.16 mm	0.21 mm	0.15 mm	0.16 mm
Type locality	Nanji Islands on East China Sea	western India	Lombok	Torres Strait	Luzon	Mergui Archipelago	Sri Lanka	Torres Strait
Distribution	Western Pacific	Indian Ocean	Western Pacific	Western Pacific	Western Pacific	Indian Ocean	Indian Ocean	Western Pacific
Depth (m)	14	6–8	22	12–37	–	–	–	–
References	present study	Williams and Vennam 2001; Williams and Chen 2012; Ramvilas 2023	Nutting 1910; Grasshoff and Alderslade 1997	Ridley 1884; Thomson and Henderson 1905; Kükenthal 1919, 1924; Williams and Vennam 2001	Kükenthal 1919; Grasshoff and Alderslade 1997	Ridley 1888; Kükenthal 1924	Thomson and Henderson 1905; Williams and Vennam 2001; Williams and Chen 2012; Ramvilas 2023	Wright and Studer 1889; Williams and Vennam 2001

##### ﻿Genetic distance and phylogenetic analyses

The new sequences were deposited in GenBank (Table 1). The alignments comprised 720, 575 and 717 nucleotide positions for the mtMutS, COI, and 28S rDNA regions, respectively. Based on the aligned region of mtMutS, the interspecific distance of *P.australiensis* Ridley, 1884, *P.fredericki*, *P.nanjiensis* sp. nov. and *P.rubrotincta* Thomson & Henderson, 1905 ranged from zero to 0.44%, while the intraspecific distance was zero, which was calculated from only two specimens of *P.nanjiensis* sp. nov. (Suppl. material 1: table S1). The mtMutS genetic distances between *P.nanjiensis* sp. nov. and congeners are in the range of 0–0.28%. For the 28S rDNA and COI regions, only sequences of *P.nanjiensis* sp. nov. are currently available within the genus *Pseudopterogorgia*, and no genetic variation was detected between the two specimens analyzed (Suppl. material 1: tables S2, S3). The genetic distances between *Pseudopterogorgia* and other genera of Gorgoniidae are greater than 5.48% for mtMutS, 5.74% for 28S, and 2.04% for COI (Suppl. material 1: tables S1–S3).

**Figure 4.**
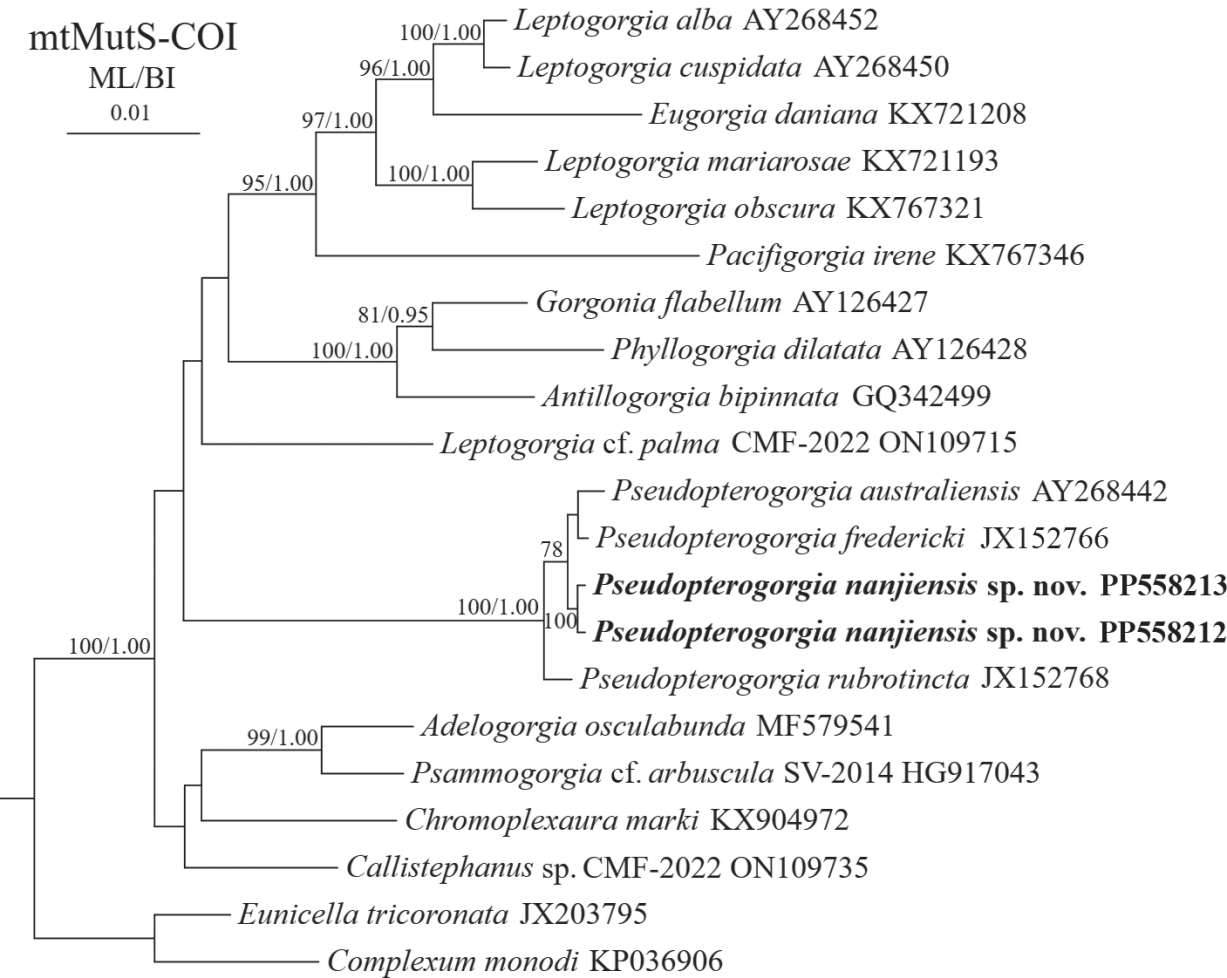
Bayesian inference (BI) tree constructed by mtMutS and COI showing phylogenetic relationships among *Pseudopterogorgia* and related genera and species. The maximum likelihood (ML) tree is identical to the BI tree in topology. Node support is as follows: ML bootstrap/BI posterior probability. The ML bootstrap < 70% or BI posterior probability < 0.95 is not shown. Newly sequenced species are in bold. The GenBank accession numbers of the mtMuts sequences are listed next to the species names.

**Figure 5.**
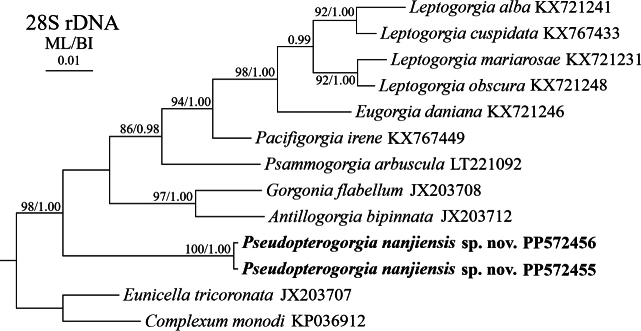
Bayesian inference (Bl) tree constructed by 28S rDNA showing phylogenetic relationships among *Pseudopterogorgia* and related genera and species. The maximum likelihood (ML) tree is identical to the BI tree in topology. Node support is as follows: ML bootstrap/BI posterior probability. The ML bootstrap < 70% or BI posterior probability < 0.95 is not shown. Newly sequenced species are in bold. The GenBank accession numbers of the 28S rDNA sequences are listed next to the species names.

The Bayesian inference (BI) tree is nearly identical to the maximum likelihood (ML) tree in topology for both the mtMutS-COI and 28S rDNA regions, and thus only the BI tree annotated with support values from both inference methods is presented (Figs 4, 5). In the mtMutS-COI trees, all the *Pseudopterogorgia* species formed a monophyletic clade with full node support. The new species *P.nanjiensis* sp. nov. clustered with the clade comprising *P.fredericki* and *P.australiensis* (Fig. 4). In the 28S rDNA trees, *P.nanjiensis* sp. nov. emerged as a distinct clade, with a basal branching position within the family Gorgoniidae (Fig. 5).

## ﻿Discussion

The genus *Pseudopterogorgia* is distinguished by its pinnate or irregular branching structures, absence of anastomoses, and C-shaped or scaphoid-like sclerites present in the coenenchyme (Kükenthal 1919; Bayer 1961; Grasshoff and Alderslade 1997). Based on the branching structure and sclerite form, the present species is undoubtedly assigned to this genus. In the mtMutS-COI trees, *Pseudopterogorgiananjiensis* sp. nov. showed a phylogenetic affinity with *P.fredericki* and *P.australiensis*, congruent with morphological assessments (Fig. 4). All three species are characterized by the absence of anastomoses and the presence of scaphoid-like sclerites within the coenenchyme. However, *P.nanjiensis* sp. nov. can be differentiated from *P.fredericki* based on sclerite morphology (see above remarks), and it is easily separated from *P.australiensis* by the branching form (irregular vs. pinnate/plumose; Table 2; Ridley 1884; Williams and Vennam 2001). With the addition of the new species *P.nanjiensis* sp. nov. discovered on the Nanji Islands, there are currently a total of eight valid species in *Pseudopterogorgia* (Williams and Vennam 2001; WoRMS Editorial Board 2024).

In the present study, the precise phylogenetic placement of the genus *Pseudopterogorgia* remains vague, primarily attributed to the low support value assigned to the corresponding node in the mtMutS-COI trees (Fig. 4). Although the 28S rDNA trees exhibit high support values for the majority of internal branches, the systematic position of *Pseudopterogorgia* remains unresolved (Fig. 5). This uncertainty arises from the inclusion of sequences representing only seven out of a total of thirteen genera currently recognized within the family Gorgoniidae, and is further compounded by the acknowledged reality that many of these genera exhibit polyphyletic or paraphyletic relationships (Vargas et al. 2014; Poliseno et al. 2017). Therefore, to achieve a clearer resolution, further genomic data are essential. These data should encompass a more exhaustive collection of 28S rDNA sequences and potentially include ultra-conserved elements plus exon (UCEs+exon).

## Supplementary Material

XML Treatment for Pseudopterogorgia nanjiensis

## ﻿Additional information

### Author ORCIDs

Yu Xu https://orcid.org/0000-0002-4937-122X

Zifeng Zhan https://orcid.org/0000-0003-4386-0687

### Data availability

All of the data that support the findings of this study are available in the main text or Supplementary Information.

## Supplementary materials

Supplementary material 1 Genetic distance analyses Data type docx Explanation note **table S1.** Interspecific and intraspecific uncorrected pairwise distances at mtMutS of *Pseudopterogorgia* and species of other genera within Gorgoniidae. **table S2.** Interspecific and intraspecific distances at 28S of *Pseudopterogorgia* and species of other genera within Gorgoniidae. **table S3.** Interspecific and intraspecific distances at COI of *Pseudopterogorgia* and species of other genera within Gorgoniidae. https://binary.pensoft.net/file/1142669This dataset is made available under the Open Database License (http://opendatacommons.org/licenses/odbl/1.0/). The Open Database License (ODbL) is a license agreement intended to allow users to freely share, modify, and use this Dataset while maintaining this same freedom for others, provided that the original source and author(s) are credited.Tingzai Sun, Yu Xu, Kuidong Xu, Shun Chen, Shangwei Xie, Zifeng Zhan
