# Supplementary material for: ﻿A new gorgonian Pseudopterogorgiananjiensis sp. nov. (Cnidaria, Octocorallia, Gorgoniidae) from the Nanji Islands, China
Source: Zookeys. 2024 Sep 27;1213:237–49. doi: 10.3897/zookeys.1213.126841 (PMC11452742; doi:10.3897/zookeys.1213.126841)
Supplement: Supplementary material 1 — Genetic distance analyses [file zookeys-1213-237_article-126841__-s001.docx]

**Supplementary material**

**Table S1.** Interspecific and intraspecific uncorrected pairwise distances at mtMutS of *Pseudopterogorgia* and species of other genera within Gorgoniidae.

|  | **Species/populations** | **1** | **2** | **3** | **4** | **5** | **6** | **7** | **8** | **9** | **10** | **11** | **12** | **13** | **14** |
| --- | --- | --- | --- | --- | --- | --- | --- | --- | --- | --- | --- | --- | --- | --- | --- |
| 1 | ***Pseudopterogorgia nanjiensis* sp. nov. PP558212, PP558213** | 0 |  |  |  |  |  |  |  |  |  |  |  |  |  |
| 2 | *Pseudopterogorgia fredericki* JX152766 | 0 | - |  |  |  |  |  |  |  |  |  |  |  |  |
| 3 | *Pseudopterogorgia australiensis* AY268442 | 0.16% | 0.15% | - |  |  |  |  |  |  |  |  |  |  |  |
| 4 | *Pseudopterogorgia rubrotincta* JX152768 | 0.28% | 0.26% | 0.44% | - |  |  |  |  |  |  |  |  |  |  |
| 5 | *Leptogorgia* cf. *palma* CMF-2022 ON109715 | 5.59% | 5.48% | 5.62% | 5.48% | - |  |  |  |  |  |  |  |  |  |
| 6 | *Leptogorgia obscura* KX767321 | 6.54% | 6.22% | 5.79% | 6.22% | 4.87% | - |  |  |  |  |  |  |  |  |
| 7 | *Leptogorgia mariarosae* KX721193 | 6.71% | 6.24% | 5.96% | 6.24% | 5.18% | 1.19% | - |  |  |  |  |  |  |  |
| 8 | *Leptogorgia cuspidata* AY268450 | 7.41% | 6.86% | 6.26% | 6.86% | 5.32% | 2.39% | 2.67% | - |  |  |  |  |  |  |
| 9 | *Leptogorgia alba* AY268452 | 6.96% | 6.44% | 5.82% | 6.44% | 4.90% | 1.97% | 2.26% | 0.42% | - |  |  |  |  |  |
| 10 | *Antillogorgia bipinnata* GQ342499 | 6.69% | 6.50% | 6.09% | 6.50% | 5.17% | 5.44% | 6.06% | 5.93% | 5.51% | - |  |  |  |  |
| 11 | *Phyllogorgia dilatata* AY126428 | 7.05% | 6.85% | 6.91% | 6.85% | 6.28% | 6.54% | 6.71% | 6.86% | 6.45% | 2.95% | - |  |  |  |
| 12 | *Gorgonia flabellum* AY126427 | 6.39% | 6.08% | 5.77% | 6.08% | 5.81% | 6.23% | 6.55% | 6.55% | 6.14% | 2.80% | 2.61% | - |  |  |
| 13 | *Pacifigorgia irene* KX767346 | 6.20% | 5.91% | 5.61% | 5.91% | 5.31% | 4.06% | 4.20% | 4.12% | 3.70% | 6.02% | 6.51% | 6.35% | - |  |
| 14 | *Eugorgia daniana* KX721208 | 7.62% | 7.07% | 7.02% | 6.92% | 6.21% | 2.58% | 2.87% | 3.01% | 2.59% | 7.39% | 8.05% | 7.41% | 5.12% | - |

**Table S2.** Interspecific and intraspecific distances at 28S of *Pseudopterogorgia* and species of other genera within Gorgoniidae.

|  | **Species/populations** | **1** | **2** | **3** | **4** | **5** | **6** | **7** | **8** | **9** | **10** |
| --- | --- | --- | --- | --- | --- | --- | --- | --- | --- | --- | --- |
| 1 | ***Pseudopterogorgia nanjiensis* sp. nov. PP572455, PP572456** | 0 |  |  |  |  |  |  |  |  |  |
| 2 | *Leptogorgia obscura* KX721248 | 7.82% | - |  |  |  |  |  |  |  |  |
| 3 | *Leptogorgia mariarosae* KX721231 | 7.68% | 0.77% | - |  |  |  |  |  |  |  |
| 4 | *Leptogorgia cuspidata* KX767433 | 7.64% | 2.12% | 2.13% | - |  |  |  |  |  |  |
| 5 | *Leptogorgia alba* KX721241 | 8.48% | 2.32% | 2.60% | 0.98% | - |  |  |  |  |  |
| 6 | *Antillogorgia bipinnata* JX203712 | 5.74% | 5.21% | 4.22% | 6.39% | 6.22% | - |  |  |  |  |
| 7 | *Gorgonia flabellum* JX203708 | 5.90% | 5.23% | 4.81% | 5.60% | 5.36% | 2.28% | - |  |  |  |
| 8 | *Psammogorgia arbuscula* LT221092 | 8.24% | 4.95% | 5.59% | 4.98% | 4.78% | 5.74% | 5.11% | - |  |  |
| 9 | *Pacifigorgia irene* KX767449 | 7.18% | 2.99% | 2.87% | 2.84% | 3.12% | 5.38% | 5.23% | 3.72% | - |  |
| 10 | *Eugorgia daniana* KX721246 | 8.36% | 2.33% | 2.74% | 2.70% | 2.85% | 6.10% | 5.39% | 4.18% | 2.47% | - |

**Table S3.** Interspecific and intraspecific distances at COI of *Pseudopterogorgia* and species of other genera within Gorgoniidae.

|  | **Species/populations** | **1** | **2** | **3** | **4** | **5** | **6** | **7** | **8** | **9** | **10** |
| --- | --- | --- | --- | --- | --- | --- | --- | --- | --- | --- | --- |
| 1 | ***Pseudopterogorgia nanjiensis* sp. nov. PP556906, PP556907** | 0 |  |  |  |  |  |  |  |  |  |
| 2 | *Leptogorgia* cf. *palma* MW401657 | 2.67% | - |  |  |  |  |  |  |  |  |
| 3 | *Leptogorgia obscura* KX767383 | 3.57% | 2.92% | - |  |  |  |  |  |  |  |
| 4 | *Leptogorgia mariarosae* KX721174 | 3.03% | 2.45% | 0.87% | - |  |  |  |  |  |  |
| 5 | *Leptogorgia cuspidata* HG917088 | 2.48% | 1.74% | 1.74% | 1.30% | - |  |  |  |  |  |
| 6 | *Leptogorgia alba* HG917083 | 2.66% | 1.86% | 1.85% | 1.41% | 0.11% | - |  |  |  |  |
| 7 | *Antillogorgia bipinnata* MK153463 | 2.17% | 2.01% | 2.49% | 2.01% | 1.70% | 1.85% | - |  |  |  |
| 8 | *Gorgonia flabellum* GQ342418 | 2.04% | 1.77% | 1.77% | 1.54% | 1.30% | 1.42% | 0.63% | - |  |  |
| 9 | *Pacifigorgia irene* KX767406 | 4.69% | 4.13% | 4.10% | 3.64% | 3.20% | 3.31% | 3.60% | 3.72% | - |  |
| 10 | *Eugorgia daniana* KX721189 | 2.85% | 2.81% | 2.74% | 2.30% | 0.98% | 1.08% | 2.17% | 2.38% | 4.22% | - |
